# A new species of the genus *Petrobia* Murray (Prostigmata, Tetranychidae) and complementary description of *Petrobiacardi* Chaudhri

**DOI:** 10.3897/BDJ.13.e141566

**Published:** 2025-02-10

**Authors:** Muhammad Kamran, Fahad Jaber Alatawi

**Affiliations:** 1 Department of Plant Protection, College of Food and Agriculture Sciences, King Saud University, Riyadh, Saudi Arabia Department of Plant Protection, College of Food and Agriculture Sciences, King Saud University Riyadh Saudi Arabia

**Keywords:** *Tetranychina*, *pakistanensis*, male, taxonomy, biodiversity

## Abstract

**Background:**

A new species, Petrobia (Petrobia) pakistanensis sp. nov. is described and collected, based on females form wild grasses, from Pakistan. the species P. (Tetranychina) cardi Chaudhri, W.M., 1972 is re-described and illustrated, based on femles and male.

**New information:**

Petrobia (Petrobia) pakistanensis sp. nov. (Prostigmata, Tetranychidae) is described and illustrated, based on female specimens collected from wild grasses (Poaceae) from Azad Kashmir, Pakistan. The male of P. (Tetranychina) cardi Chaudhri, reported for the first time, is described and illustrated. Additionally, the complementary description of females of this species is provided, based on a new collection.

## Introduction

The genus *Petrobia* Murray (Acari, Prostigmata,Tetranychidae), the most diverse amongst the genera of the tribe Petrobiini Reck, comprises three subgenera, *Petrobia* Murray (19 spp.), *Tetranychina* Reck (15 spp.) and *Mesotetranychus* Banks (9 spp.) (Mirza et al. 2023, Migeon and Dorkeld 2024). These species are polyphagous found on economically important plant species and wild vegetation (Jeppson et al. 1975,Bolland et al. 1998) and distributed all over the world (Khanjani et al. 2016, Migeon and Dorkeld 2024). A few species are considered severe pests of some economic crops and are known to transmit viruses Robertson and Brumfield 2000).

Previously, eight *Petrobia* species *Petrobia (P.) latens, P*. (Tetranychina) *nocitus* Chaudhri, P. (T.) cardi Chaudhri, P. (T.) tribulus Chaudhri, P. (T.) nocitus Chaudhri, P. (T.) afzali Sabri and Afzal, P. (T.) chaudhrii Sabri and Afzal and P. (T.) kleptes Kamran and Afzal, have been reported from different regions of Pakistan.

The recent survey of the Northern Region of Pakistan (Azad Kashmir) resulted in the identification of two *Petrobia* species, P. (P.) pakistanensis sp. nov. and P. (Tetranychina) cardi Chaudhri found inhabiting grasses (Poaceae). In this study, the new species is described and illustrated from female specimens. Moreover, the male of *P.cardi* is described and illustrated for the first time along with a complementary description of *P.cardi*.

## Materials and methods

The mite specimens were collected from the host plant foliage by shaking the plant parts over a white paper sheet. The preserved mite specimens were directly mounted on glass slides using Hoyer’s medium under a stereomicroscope (SZX10, Olympus, Tokyo, Japan) and identified under a phase contrast microscope (DM2500, Lieca, Wetzlar, Germany) using the published identification key (Martinez et al. 2014, Khanjani et al. 2016). Different body parts were then pictured using an auto-montage software system (Syncroscopy, Cambridge, UK) and illustrated using Adobe Illustrator (Adobe System Inc., San Jose, CA, USA). The terminology and leg chaetotaxy followed those of Lindquist (1985). All measurements of the holotype and paratypes (presented as ranges in parentheses) for the new species are given in micrometres (μm). Body dimensions were measured as: the length between setae *v*_2_–*h*_1_ and the width between *c*_3_–*c*_3_; setae were measured from their insertion to their tips; and distance between setae as the distance between their insertions. Legs were measured from the base of trochanter to the tip of pretarsus (bases of claws and empodium).

Tarsal setae counts are presented as total number of tactile setae including: eupathidia, number of solenidia and duplex setae. All specimens including type specimens of the new species were deposited in the King Saud University Museum of Arthropods (KSMA), Acarology Section, Department of Plant Protection, College of Food and Agriculture Sciences, King Saud University, Riyadh, Saudi Arabia.

## Taxon treatments

### Petrobia (Petrobia) pakistanensis

Kamran & Alatawi sp. nov.

147807CE-F792-5477-8577-EEAAA5251332

6B5E72E3-C444-4BD9-AEF3-E9C1AD94F78A

#### Materials

- **Type status:** Holotype. **Occurrence:** catalogNumber: KSMAAS-23-Tet-Pet-H; recordedBy: M. Kamran; individualCount: 1; sex: Female; lifeStage: adult; occurrenceID: 8B0FBB5F-8941-5BA7-B297-3E300A8842F4; **Taxon:** scientificName: Petrobiapakistanensis; kingdom: Animalia; phylum: Arthropoda; class: Arachnida; order: Prostigmata; family: Tetranychidae; genus: Petrobia; subgenus: Petrobia; **Location:** country: Pakistan; stateProvince: Khyber Pakhtunkhwa; locality: Abbotabad; verbatimElevation: 1,256 m; verbatimCoordinates: 34°08.427'N 73°17.135'E; decimalLatitude: 34.14046; decimalLongitude: 73.28559; georeferenceProtocol: GPS; **Identification:** identifiedBy: Muhammad Kamran; dateIdentified: 2023; **Event:** samplingProtocol: shaking plant foliage; eventDate: 15/7/2023; habitat: wild grasses; **Record Level:** language: en; collectionCode: Mites; basisOfRecord: Slide Mounted Specimen
- **Type status:** Paratype. **Occurrence:** catalogNumber: KSMAAS-23-Tet-Pet-P1; recordedBy: M. Kamran; individualCount: 1; sex: Female; lifeStage: adult; occurrenceID: 4EDE717B-64C6-58D4-A4E1-883A75981821; **Taxon:** scientificName: Petrobiapakistanensis; kingdom: Animalia; phylum: Arthropoda; class: Arachnida; order: Prostigmata; family: Tetranychidae; genus: Petrobia; subgenus: Petrobia; **Location:** country: Pakistan; stateProvince: Khyber Pakhtunkhwa; locality: Abbotabad; verbatimElevation: 1,256 m; verbatimCoordinates: 34°08.427'N 73°17.135'E; decimalLatitude: 34.14046; decimalLongitude: 73.28559; georeferenceProtocol: GPS; **Identification:** identifiedBy: Muhammad Kamran; dateIdentified: 2023; **Event:** samplingProtocol: shaking plant foliage; eventDate: 15/7/2023; habitat: wild grasses; **Record Level:** language: en; collectionCode: Mites; basisOfRecord: Slide Mounted Specimen
- **Type status:** Paratype. **Occurrence:** catalogNumber: KSMAAS-23-Tet-Pet-P2; recordedBy: M. Kamran; individualCount: 1; sex: Female; lifeStage: adult; occurrenceID: E4BED139-7480-58C2-AEAF-502CB0507B02; **Taxon:** scientificName: Petrobiapakistanensis; kingdom: Animalia; phylum: Arthropoda; class: Arachnida; order: Prostigmata; family: Tetranychidae; genus: Petrobia; subgenus: Petrobia; **Location:** country: Pakistan; stateProvince: Khyber Pakhtunkhwa; locality: Abbotabad; verbatimElevation: 1,256 m; verbatimCoordinates: 34°08.427'N 73°17.135'E; decimalLatitude: 34.14046; decimalLongitude: 73.28559; georeferenceProtocol: GPS; **Identification:** identifiedBy: Muhammad Kamran; dateIdentified: 2023; **Event:** samplingProtocol: shaking plant foliage; eventDate: 15/7/2023; habitat: wild grasses; **Record Level:** language: en; collectionCode: Mites; basisOfRecord: Slide Mounted Specimen
- **Type status:** Paratype. **Occurrence:** catalogNumber: KSMAAS-23-Tet-Pet-P3; recordedBy: M. Kamran; individualCount: 1; sex: Female; lifeStage: adult; occurrenceID: 9F29037F-B50F-5CEB-8DB4-F048FADCD2FF; **Taxon:** scientificName: Petrobiapakistanensis; kingdom: Animalia; phylum: Arthropoda; class: Arachnida; order: Prostigmata; family: Tetranychidae; genus: Petrobia; subgenus: Petrobia; **Location:** country: Pakistan; stateProvince: Khyber Pakhtunkhwa; locality: Abbotabad; verbatimElevation: 1,256 m; verbatimCoordinates: 34°08.427'N 73°17.135'E; decimalLatitude: 34.14046; decimalLongitude: 73.28559; georeferenceProtocol: GPS; **Identification:** identifiedBy: Muhammad Kamran; dateIdentified: 2023; **Event:** samplingProtocol: shaking plant foliage; eventDate: 15/7/2023; habitat: wild grasses; **Record Level:** language: en; collectionCode: Mites; basisOfRecord: Slide Mounted Specimen
- **Type status:** Paratype. **Occurrence:** catalogNumber: KSMAAS-23-Tet-Pet-P4; recordedBy: M. Kamran; individualCount: 1; sex: Female; lifeStage: adult; occurrenceID: AA115F81-79C9-5DE0-A75D-F2FA9A951D75; **Taxon:** scientificName: Petrobiapakistanensis; kingdom: Animalia; phylum: Arthropoda; class: Arachnida; order: Prostigmata; family: Tetranychidae; genus: Petrobia; subgenus: Petrobia; **Location:** country: Pakistan; stateProvince: Khyber Pakhtunkhwa; locality: Abbotabad; verbatimElevation: 1,256 m; verbatimCoordinates: 34°08.427'N 73°17.135'E; decimalLatitude: 34.14046; decimalLongitude: 73.28559; georeferenceProtocol: GPS; **Identification:** identifiedBy: Muhammad Kamran; dateIdentified: 2023; **Event:** samplingProtocol: shaking plant foliage; eventDate: 15/7/2023; habitat: wild grasses; **Record Level:** language: en; collectionCode: Mites; basisOfRecord: Slide Mounted Specimen
- **Type status:** Paratype. **Occurrence:** catalogNumber: KSMAAS-23-Tet-Pet-P5; recordedBy: M. Kamran; individualCount: 1; sex: Female; lifeStage: adult; occurrenceID: 8B0C61C0-E8FE-5F44-9020-5F53EC4E0FC9; **Taxon:** scientificName: Petrobiapakistanensis; kingdom: Animalia; phylum: Arthropoda; class: Arachnida; order: Prostigmata; family: Tetranychidae; genus: Petrobia; subgenus: Petrobia; **Location:** stateProvince: Khyber Pakhtunkhwa; locality: Abbotabad; verbatimElevation: 1,256 m; verbatimCoordinates: 34°08.427'N 73°17.135'E; decimalLatitude: 34.14046; decimalLongitude: 73.28559; georeferenceProtocol: GPS; **Identification:** identifiedBy: Muhammad Kamran; dateIdentified: 2023; **Event:** samplingProtocol: shaking plant foliage; eventDate: 15/7/2023; habitat: wild grasses; **Record Level:** language: en; collectionCode: Mites; basisOfRecord: Slide Mounted Specimen
- **Type status:** Paratype. **Occurrence:** catalogNumber: KSMAAS-23-Tet-Pet-P6; recordedBy: M. Kamran; individualCount: 1; sex: Female; lifeStage: adult; occurrenceID: C85739B4-02FA-56EF-B8EA-004161D2BCFC; **Taxon:** scientificName: Petrobiapakistanensis; kingdom: Animalia; phylum: Arthropoda; class: Arachnida; order: Prostigmata; family: Tetranychidae; genus: Petrobia; subgenus: Petrobia; **Location:** stateProvince: Khyber Pakhtunkhwa; locality: Abbotabad; verbatimElevation: 1,256 m; verbatimCoordinates: 34°08.427'N 73°17.135'E; decimalLatitude: 34.14046; decimalLongitude: 73.28559; georeferenceProtocol: GPS; **Identification:** identifiedBy: Muhammad Kamran; dateIdentified: 2023; **Event:** samplingProtocol: shaking plant foliage; eventDate: 15/7/2023; habitat: wild grasses; **Record Level:** language: en; collectionCode: Mites; basisOfRecord: Slide Mounted Specimen

#### Description

**Female** (n = 7). Length of body excluding gnathosoma (*v*_2_-*h*_1_) 510 (505‒518), including gnathosoma 695 (690–710), width (*c*_3_-*c*_3_) 410(400‒517); length of legs (from trochanter to distal end of tarsus): leg I 444 (441‒454); leg II 303 (300‒314); leg III 334 (327‒340); leg IV 391 (382‒399); leg I/*v*_2_-*h*_1_: 0.87 (0.85–0.87).

**Figure 1.**
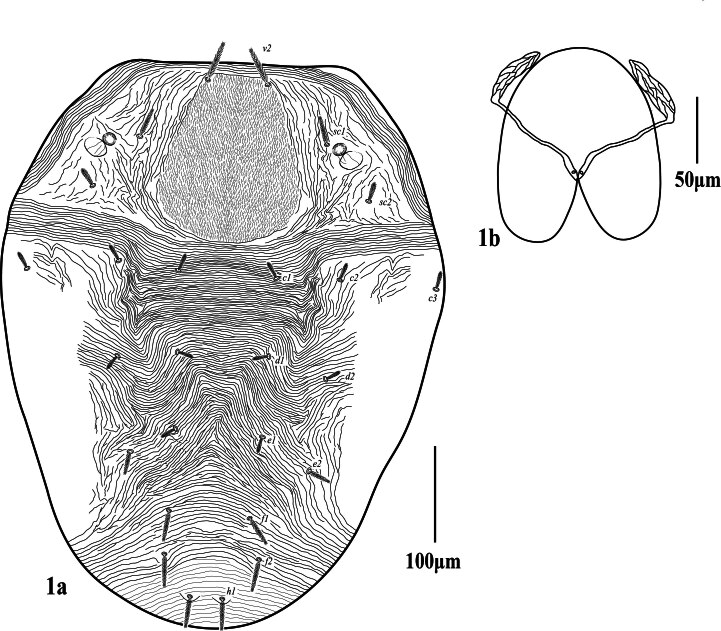
Petrobia (Petrobia) pakistanensis sp. nov. Female, 1a dorsum. Scale bar 100 μm; 1b Stylophore. Scale bar 50 μm.

**Dorsum.** (Fig. 1) Propodosomal shield well defined, punctate, two pairs of eyes; lateral propodosoma with longitudinal to oblique striations, opisthosomal striae transverse medially between setae *c*_1_-*h*_1_, except sub-medially changing to inverted v-shaped striation pattern in-between *e*_2_, dorsal striae fine without lobes and close (not so widely spaced). Dorsal setae lanceolate, slender serrate, without tubercles, shorter than the longitudinal distance between the setae next in line; lengths of dorsal setae *v*_2_ 42 (39‒45), *sc*_1_ 27 (26‒30), *sc*_2_ 18 (17‒20), *c*_1_ 17 (17‒18), *c*_2_ 17 (17‒18), *c*_3_ 18 (18‒19), *d*_1_ 14 (13‒14), *d*_2_ 15 (14‒15), *e*_1_ 15 (14‒16), *e*_2_ 23 (21‒24), *f*_1_ 33 (30‒34), *f*_2_ 33 (31‒33), *h*_1_ 30 (29‒31). Distances between dorsal setae *v*_2_-*h*_1_ 510 (505‒518), *v*_2_-*v*_2_ 70 (68‒71), *sc*_1_-*sc*_1_ 185 (171‒188), *sc*_2_-*sc*_2_ 285 (277‒295), *c*_1_-*c*_1_ 95 (92‒103), *c*_1_-*c*_2_ 64 (61‒65), *d*_1_-*d*_1_ 88 (82–90), *d*_1_-*d*_2_ 64 (59‒66), *e*_1_-*e*_1_ 85 (81–87), *e*_1_-*e*_2_ 54 (50‒57), *f*_1_-*f*_1_ 80 (77‒85), *f*_2_-*f*_2_ 70 (68‒76), *f*_1_-*f*_2_ 33 (33‒38), *h*_1_-*h*_1_ 35 (34–38), *c*_1_-*d*_1_ 77 (75‒81), *d*_1_-*e*_1_ 78 (76‒79), *e*_1_-*f*_1_ 82 (78‒86), *f*_2_-*f*_2_ 39 (38‒43), *f*_2_-*h*_1_ 49 (48‒53), *f*_1_-*h*_1_ 78 (75‒79).

**Figure 2.**
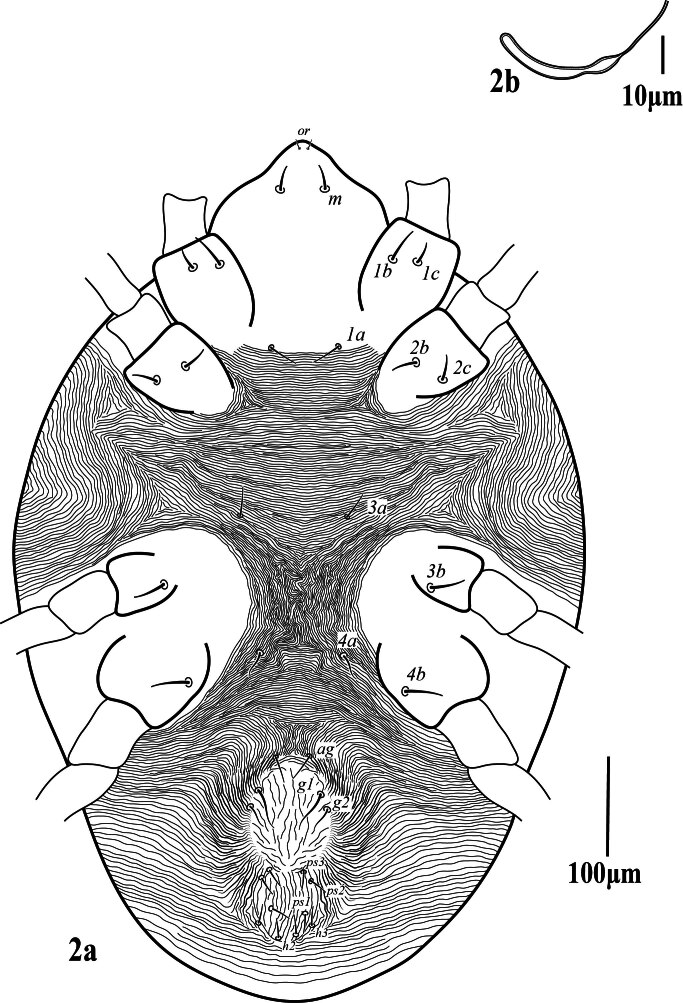
Petrobia (Petrobia) pakistanensis sp. nov. Female, 2a venter. Scale bar 100 μm; 2b spermatheca. Scale bar 10 μm.

**Venter** (Fig. 2). Area between setae _1_*a*–_3_*a* with transverse striae, _3_*a*–_4_*a* with longitudinal striae, anterior of genito-anal area with longitudinal striae; striae on venter without lobe; lengths of setae: _1_*a* 31 (30‒33), _1_*b* 40 (39–43), _1_*c* 22 (21‒23), _2_*b* 25 (25‒27), _2_*c* 25 (23‒28), _3_*a* 32 (30‒35), _3_*b* 21 (20‒24), _4_*a* 28 (27‒31), _4_*b* 20 (19‒22), one pair of aggenital setae (*ag*) 31 (27‒31), two pairs of genital setae, *g*_1_ 43 (40‒45), *g*_2_ 26 (26‒27), and three pairs of anal setae (*ps*_1‒3_), *ps_1_* 20 (20‒21), *ps*_2_ 19 (18‒20), *ps*_3_ 21 (20‒21), *h*_2_ 18 (18‒19), *h*_3_ 17 (17‒18). Spermatheca elongated tubular (Fig. 2b).

**Gnathosoma.** Stylophore (Fig. 1b) anteriorly without indentation, peritremes distally with long anastomosed ends, 47 (45‒48) long, 14 (13‒14) wide), ventral infracapitulum with one pair of adoral setae or 16 (15‒16) and one pair of subcapitular setae *m* 26 (25‒27). Palp five-segmented (Fig. 4), palptarsus with one solenidion *ω*_7_ (5‒7), three eupathidia *ul''ζ* 8 (8‒10), *ul'''ζ* 8 (7‒9), *sul* 9 (8‒11) and three simple setae *a* 13 (10‒13), *b* 12 (10‒13); *c* 11 (11‒12); palp tibia with three setae *d* 23 (19‒23), *l*'' 16 (15‒18), *l*' 17 (16‒19) and one bifid claw distally, palp genu with one simple seta *l*'' 28 (24‒28) and palp femur with one serrated seta *d* 34 (32‒36) (similar to dorsal setae).

**Figure 3.**
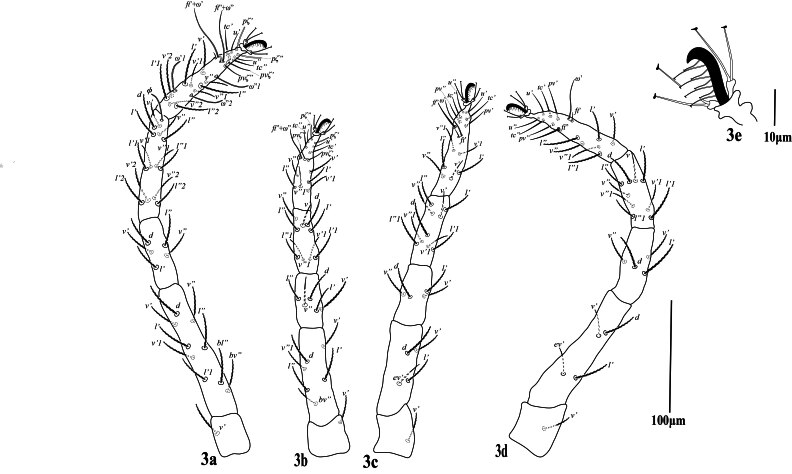
Petrobia (Petrobia) pakistanensis sp. nov. Female, **a3** Leg I; **b3** Leg II; **c3** Leg III; **d3** Leg IV. Scale bar 100 μm; **3e** Empodium IV. Scale bar 10 μm.

**Figure 4.**
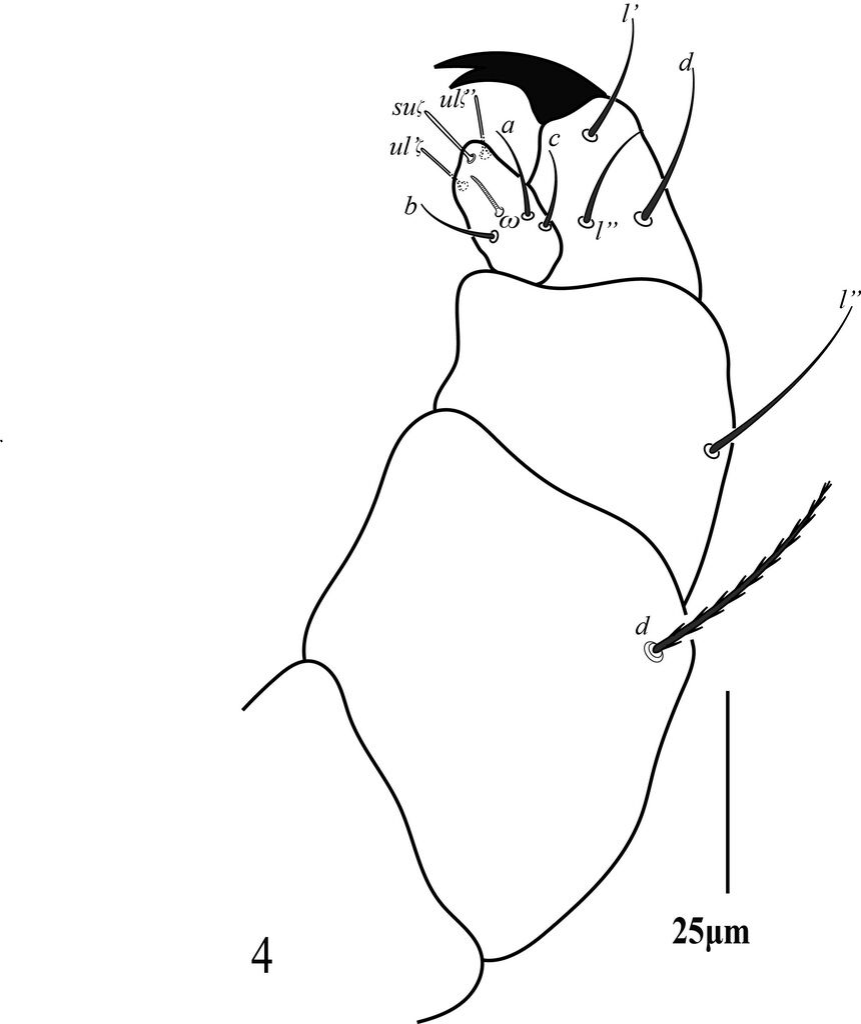
Petrobia (Petrobia) pakistanensis sp. nov. Female, palp. Scale bar 25 μm.

**Legs** (Fig. 3). Leg I (trochanter to distal end of empodial claw) shorter than length of idiosoma. Leg segment setal formula as follows: coxae 2-2-1-1; trochanters 1-1-1-1; femora 9-6-4-4; genua 5-5-4-4; tibiae 13+1φ)-9-9-9; tarsi 18 +3 ω+2dup-14 +1dup-13 +1dup-13 +1dup. Length of solenidia on tarsi I ‒IV: Iω'_1_ 32 (30‒34), Iω'67 (66‒70), Iω" 75 (74‒78), IIω” 47 (45‒48), IIIω’ 45 (43‒46), IV ω’ 46 (45‒47) and tibia I with Iφ1 26 (25‒26). Most leg setae narrowly lanceolate, barbed; setae at tips of tarsi slender, smooth. Empodia I–IV with true claws pad-like, empodial claws bearing two rows of ventrally directed hairs.

Male and immature stages—Unknown.

#### Diagnosis

Propodosomal shield punctate; dorsal body setae lanceolate, barbed, short: setae *c*_1_, *d*_1_, *e*_1_ not reaching beyond half-way between setal insertion and insertion in next row; tubercles absent, opisthosomal striae fine, smooth, without lobes and close; dorsal opisthosoma forming inverted v-shaped striation pattern between setae *e*; seta *h*_1_ set on small tubercles; peritremes distally anastomosed, anastomosed part 44‒47 long and 13‒14 wide. Leg I (trochanter to distal end of tarsus) shorter than length of idiosoma; setal formula on leg segments: femora 9-6-4-4; genua 5-5-4-4; tibiae 13(1φ)- 9-9-9; tarsi 18(1ω + 2dup) -14(1dup)-13(1dup)-13(1dup).

#### Etymology

The new species is named after the name of country “Pakistan”, where the type specimens were collected.

#### Remarks

*Petrobiapakistanensis* sp. nov. resembles *P.haematoxylon* Meyer, 1987 and *P.norbakhshi* Khanjani, Khanjani & Seeman, 2016 (Meyer 1987 and Khanjani et al. 2016), by having the same setal formulae of coxae to femora and striation pattern on dorsum. However, *P.pakistanensis* sp. nov. differs from *P.haematoxylon* in: dorsal opisthosoma forming inverted v-shaped striation pattern between setae *e*_1_ in the new species compared to transverse striae in *P.haematoxylon*; anastomosed part of peritremes more than three times as long as wide in the new species than as long as wide in *P.haematoxylon*; seta *h*_1_ on small tubercles in new species compared seta *h*_1_ without tubercles in *P.haematoxylon* and setae on tibia II and III each with nine setae versus 7 or 8 setae in *P.haematoxylon.* Additionally, *P.pakistanensis* sp. nov. differs from *P.norbakhshi* by propodosoma medially punctate compared to pebbled pattern in *P.norbakhshi*; genu III and IV each with four setae in new species compared to six setae each on genua III and IV in *P.norbakhshi*; tarsus I with 3 solenidion in new species compared to six or seven solenidion in *P.norbakhshi*; tarsus II with 14 (1dup) setae as compared to tarsus II with 15 (1ω + 1dup) setae in *P.norbakhshi*.

### Petrobia (Tetranychina) cardi

Chaudhri, W.M., 1972

D802FF4D-D812-5224-B5E9-D9DA753C6DCC

#### Materials

- **Type status:** Other material. **Occurrence:** catalogNumber: KSMAAS-23-Tet-Pet-01-Species record; recordedBy: M. Kamran; individualCount: 1; sex: Female; lifeStage: adult; occurrenceID: B47E3CCE-28BE-57E4-B979-A83473BC0A25; **Taxon:** scientificName: Petrobiacardi; nameAccordingTo: Chaudhri, W., 1972. Mites of the genus Petrobia. I. Description of three new species of mites from Pakistan (Tetranychidae); kingdom: Animalia; phylum: Arthropoda; class: Arachnida; order: Prostigmata; family: Tetranychidae; genus: Petrobia; subgenus: Tetranychina; **Location:** country: Pakistan; stateProvince: Kashmir; verbatimCoordinates: 34°34.030'N 73°53.478'E; decimalLatitude: 34.56717; decimalLongitude: 73.8913; georeferenceProtocol: GPS; **Identification:** identifiedBy: Muhammad Kamran; dateIdentified: 2023; **Event:** samplingProtocol: shaking plant foliage; eventDate: 18/7/2023; habitat: wild grasses; **Record Level:** language: en; collectionCode: Mites; basisOfRecord: Slide Mounted Specimen
- **Type status:** Other material. **Occurrence:** catalogNumber: KSMAAS-23-Tet-Pet-02- Species record; recordedBy: M. Kamran; individualCount: 1; sex: Female; lifeStage: adult; occurrenceID: B9BE40B9-ADF8-5D3C-9F27-3E08117BA076; **Taxon:** scientificName: Petrobiacardi; nameAccordingTo: Chaudhri, W., 1972. Mites of the genus Petrobia. I. Description of three new species of mites from Pakistan (Tetranychidae); kingdom: Animalia; phylum: Arthropoda; class: Arachnida; order: Prostigmata; family: Tetranychidae; genus: Petrobia; subgenus: Tetranychina; **Location:** country: Pakistan; stateProvince: Kashmir; verbatimCoordinates: 34°34.030'N 73°53.478'E; decimalLatitude: 34.56717; decimalLongitude: 73.8913; georeferenceProtocol: GPS; **Identification:** identifiedBy: Muhammad Kamran; dateIdentified: 2023; **Event:** samplingProtocol: shaking plant foliage; eventDate: 18/7/2023; habitat: wild grasses; **Record Level:** language: en; collectionCode: Mites; basisOfRecord: Slide Mounted Specimen
- **Type status:** Other material. **Occurrence:** catalogNumber: KSMAAS-23-Tet-Pet-03- Species record; recordedBy: M. Kamran; individualCount: 1; sex: Male; lifeStage: adult; occurrenceID: 74034B49-36C1-5800-A65B-C2F672E60511; **Taxon:** scientificName: Petrobiacardi; nameAccordingTo: Chaudhri, W., 1972. Mites of the genus Petrobia. I. Description of three new species of mites from Pakistan (Tetranychidae); kingdom: Animalia; phylum: Arthropoda; class: Arachnida; order: Prostigmata; family: Tetranychidae; genus: Petrobia; subgenus: Tetranychina; **Location:** country: Pakistan; stateProvince: Kashmir; verbatimCoordinates: 34°34.030'N 73°53.478'E; decimalLatitude: 34.56717; decimalLongitude: 73.8913; georeferenceProtocol: GPS; **Identification:** identifiedBy: Muhammad Kamran; dateIdentified: 2023; **Event:** samplingProtocol: shaking plant foliage; eventDate: 18/7/2023; habitat: wild grasses; **Record Level:** language: en; collectionCode: Mites; basisOfRecord: Slide Mounted Specimen
- **Type status:** Other material. **Occurrence:** catalogNumber: KSMAAS-23-Tet-Pet-04- Species record; recordedBy: M. Kamran; individualCount: 1; sex: Female; lifeStage: adult; occurrenceID: 7657BEF8-5E9F-5D79-9B43-4E2C47188171; **Taxon:** scientificName: Petrobiacardi; nameAccordingTo: Chaudhri, W., 1972. Mites of the genus Petrobia. I. Description of three new species of mites from Pakistan (Tetranychidae); kingdom: Animalia; phylum: Arthropoda; class: Arachnida; order: Prostigmata; family: Tetranychidae; genus: Petrobia; subgenus: Tetranychina; **Location:** country: Pakistan; stateProvince: Kashmir; verbatimCoordinates: 34°37.910'N 73°55.146'E; decimalLatitude: 34.63183; decimalLongitude: 73.91911; georeferenceProtocol: GPS; **Identification:** identifiedBy: Muhammad Kamran; dateIdentified: 2023; **Event:** samplingProtocol: shaking plant foliage; eventDate: 18/7/2023; habitat: wild grasses; **Record Level:** language: en; collectionCode: Mites; basisOfRecord: Slide Mounted Specimen
- **Type status:** Other material. **Occurrence:** catalogNumber: KSMAAS-23-Tet-Pet-05- Species record; recordedBy: M. Kamran; individualCount: 1; sex: Female; lifeStage: adult; occurrenceID: 99EA115D-E88E-5B62-A1D7-55D10CEDD3DD; **Taxon:** scientificName: Petrobiacardi; nameAccordingTo: Chaudhri, W., 1972. Mites of the genus Petrobia. I. Description of three new species of mites from Pakistan (Tetranychidae); kingdom: Animalia; phylum: Arthropoda; class: Arachnida; order: Prostigmata; family: Tetranychidae; genus: Petrobia; subgenus: Tetranychina; **Location:** country: Pakistan; stateProvince: Kashmir; verbatimCoordinates: 34°37.910'N 73°55.146'E; decimalLatitude: 34.63183; decimalLongitude: 73.91911; georeferenceProtocol: GPS; **Identification:** identifiedBy: Muhammad Kamran; dateIdentified: 2023; **Event:** samplingProtocol: shaking plant foliage; eventDate: 18/7/2023; habitat: wild grasses; **Record Level:** language: en; collectionCode: Mites; basisOfRecord: Slide Mounted Specimen

#### Description

**Female** (n = 4) Body broadly oval, Length including gnathosoma 700 (690‒715); idiosoma (*v*_2_-*h*_1_) 583 (575‒590) long, *c*_3_-*c*_3_ 507(500‒515) wide; length of legs (from trochanter to distal end of tarsus): leg I 1298 (1275‒1305); leg II 570 (565‒578); leg III 664 (655‒670), leg IV 1040 (1033‒1050); leg I/v2-h1: 2.22(2.20–2.24).

**Figure 5.**
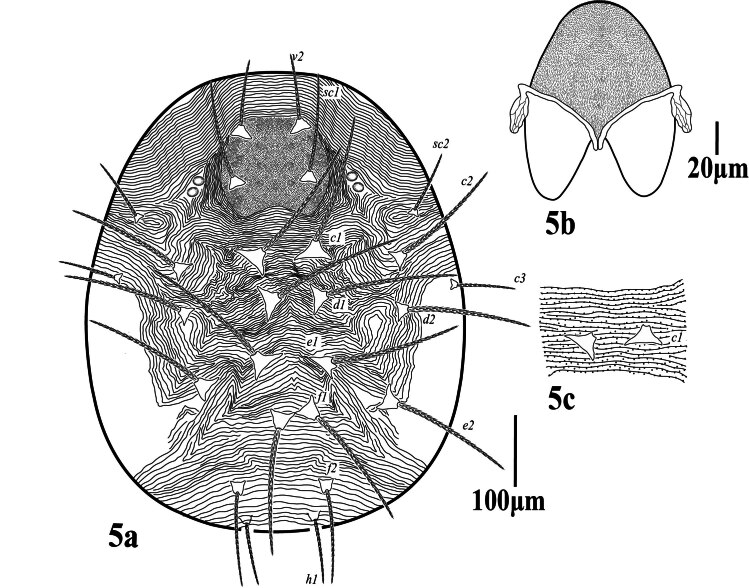
Petrobia (Tetranychina) cardi Chaudhri. Female, 5a dorsum. Scale bar 100 μm; 5b Stylophore. Scale bar 20 μm.

**Dorsum** (Fig. 5). Propodosomal shield well defined, densely punctate, two pairs of eyes; lateral propodosoma with longitudinal to oblique granulated striations, setae *sc*_1_ on prodorsal shield; opisthosomal striae transverse medially between setae *c*_1_-*h*_1_, except sub-medially changing to inverted v-shaped striation pattern just posterior to setae *d*_1_, laterally with longitudinal to oblique irregular striations, dorsal striae lobed and closely-spaced. All dorsal setae long robust, serrated and set on strong tubercles, tubercles of setae *c*_1_, *d*_1_ and *e*_1_ well apart, while of setae *f*_1_ contiguous; lengths of dorsal setae (without tubercles): *v*_2_ 95 (90‒98), *sc*_1_ 139 (130‒144), *sc*_2_ 88 (83‒90), *c*_1_ 183 (176‒185), *c*_2_ 164 (158‒169), *c*_3_ 82 (78‒88), *d*_1_ 188 (180‒193), *d*_2_ 172 (167‒179), *e*_1_ 183 (176‒188), *e*_2_ 175 (168‒177), *f*_1_ 180 (181‒190), *f*_2_ 131 (126‒138), *h*_1_ 90 (90‒97). Distances between dorsal setae *v*_2_-*h*_1_ 583 (575‒590), *v*_2_-*v*_2_ 82 (76‒85), *sc*_1_-*sc*_1_ 118 (115‒122), *sc*_2_-*sc*_2_ 355 (345‒360), *c*_1_-*c*_1_ 82 (80‒89), *c*_1_-*c*_2_ 66 (66‒69), *d*_1_-*d*_1_ 65(60–68), *d*_1_-*d*_2_ 98 (94‒99), *e*_1_-*e*_1_ 114 (110–116), *e*_1_-*e*_2_ 28 (27‒30), *f*_1_-*f*_1_ 45 (42‒47), *f*_2_-*f*_2_ 155 (150‒158), *h*_1_-*h*_1_ 98 (90–98), *c*_1_-*d*_1_ 75 (73‒78), *d*_1_-*e*_1_ 98 (93‒99), *e*_1_-*f*_1_ 85 (82‒86), *f*_1_-*f*_2_ 106 (102‒109), *f*_1_-*h*_1_ 124 (120‒126).

**Figure 6.**
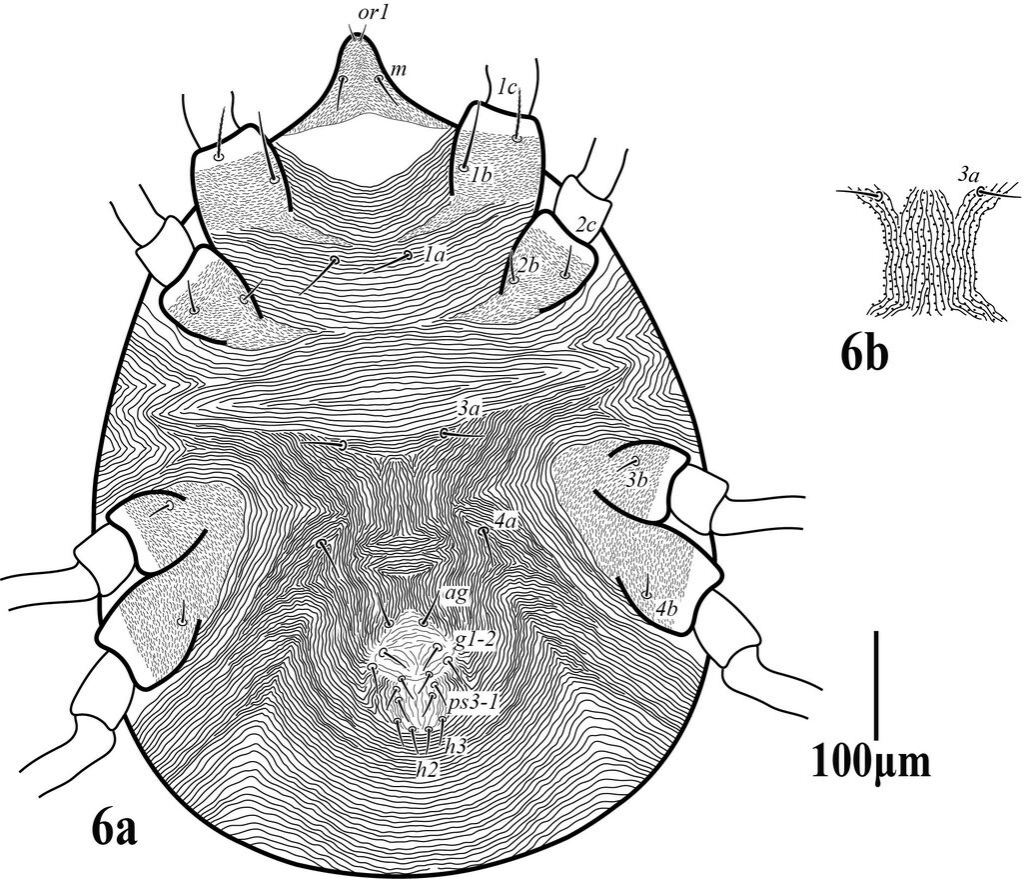
Petrobia (Tetranychina) cardi Chaudhri. Female, 6a venter. Scale bar 100 μm.

**Venter** (Fig. 6). Integument between setae _1_*a*–_3_*a* with transverse dotted striae, between _3_*a* to _4_*a* with longitudinal striae, anterior of genito-anal area with longitudinal striae. Striae on venter lobed. Lengths of setae: _1_*a* 49 (45‒49), _1_*b* 65 (55–65), _1_*c* 48 (44‒48), _2_*b* 26 (25‒27), _2_*c* 28 (24‒28), _3_*a* 37 (35‒38), _4_*a* 36 (35‒38), _3_*b* 20 (19‒20), _4_*b* 20 (19‒20), one pair of aggenital setae (*ag*) 34 (32‒34), two pairs of genital setae, *g*_1_ 26 (26‒27), *g*_2_ 26 (25‒27) and three pairs of anal setae (*ps*_1‒3_), *ps*_1_ 20 (20‒21), *ps*_2_ 19 (18‒19), *ps*3 21 (18‒21), *h*_2_ 26 (24‒26), *h*_3_ 25 (23‒25). Spermatheca elongated thread-like.

**Gnathosoma** Stylophore (Fig. 5b) anteriorly rounded, peritremes distally slightly anastomosed forming heart-shaped globular structure, 24 long, 12 wide; ventral infracapitulum with one pair of adoral setae *or* 10 (9‒10) and one pair of subcapitular setae *m* 31 (29‒31). Palp five-segmented (Fig. 8), palptarsus with one solenidion *ω* 6 (5‒8), three eupathia *ul''ζ* 8 (7‒9), *ul'ζ* 7 (6‒8), *sul* 9 (7‒9) and three setiform setae *a* 10 (9‒11), *b* 12 (11‒13), *c* 13 (10‒13); palp tibia with three setae *d* 28 (27‒30), *l*' 18 (15‒19), *l*'' 18 (17‒20) and one claw bifid distally, palp genu with one simple seta *l*'' 27 (25‒28) and palp femur with one serrated seta *d* 32 (29‒30) (similar to dorsal setae).

**Figure 7.**
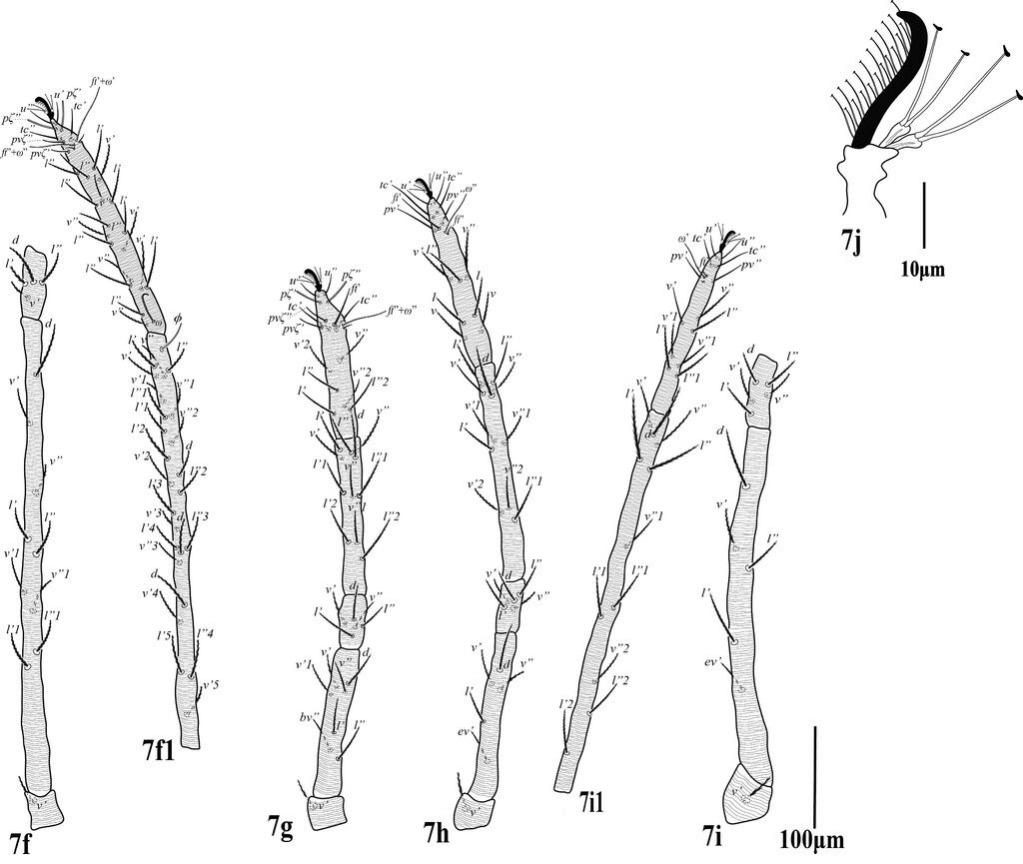
Petrobia (Tetranychina) cardi Chaudhri. Female. **f** Leg I, **7f** trochanter, femur genu; **7f1** tibia, tarsus; **7g** Leg II; **h** Leg III; **i** Leg IV, **7i** trochanter, femur, genu, **7i1** tibia, tarsus. Scale bar 100 μm. 7i Empodium IV. Scale bar 10 μm.

**Figure 8.**
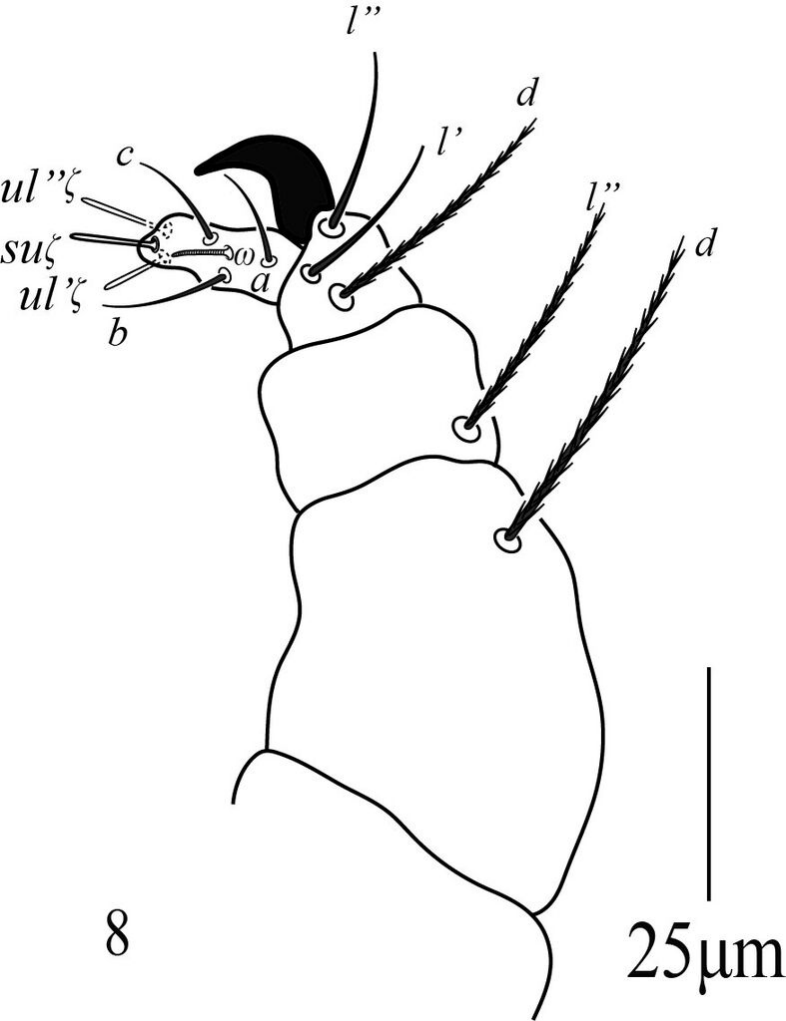
Petrobia (Tetranychina) cardi Chaudhri. Female, palp. Scale bar 25 μm.

**Legs** (Fig. 7). Leg I (trochanter to distal end of tarsus) two times longer than the length of idiosoma. Length of leg I (trochanter to tip of tarsus) 1293 (1220‒1293), leg II 570 (560‒575), leg III 660(520‒660, leg IV 1030 (981‒1032); length of leg segments, Leg I (trochanter 40‒45, femur 490‒513, genu 55‒60, tibia 470‒495, tarsus 165‒180; leg IV trochanter 50 (46‒50), femur 352 (340‒355), genu 72 (65‒75), tibia 400 (385‒400), tarsus 155 (145‒155). Leg segment setal formula as follows: coxae 2-2-1-1; trochanters 1-1-1-1; femora 9-7-5-5; genua 4-5-5-5; tibiae 24+1φ-11-11-11; tarsi 26 +1ω+2dup-15+1dup-14 +1dup-14 +1dup; solenidia on leg I Iω” 50 (48‒50), IIω” 53 (50‒53), IIIω’ 30 (29‒30), IV ω’ 31 (30‒31). Empodia I–IV with true claws pad-like, empodial claws bearing two rows of ventrally directed hairs.

**Male** (n = 1). Body oval elongate, much shorter than female, including gnathosoma (adoral setae to *h*2) 376 long, excluding gnathosoma (*v*_2_-*h*_1_) 251 long, *c*_3_-*c*_3_ 222 wide; length of legs (from trochanter to distal end of tarsus): leg I 1164; leg II 508; leg III 556, leg IV 820.

**Figure 9.**
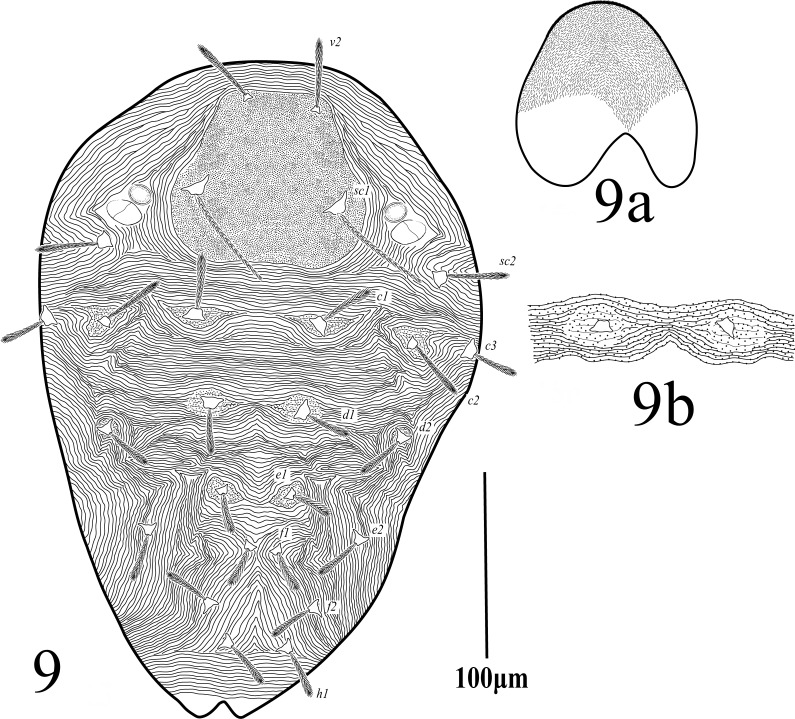
Petrobia (Tetranychina) cardi Chaudhri. Male, dorsum. Scale bar 100 μm; **9a** Stylophore. Scale bar 20 μm.

**Dorsum** (Fig. 9). Propodosomal shield well defined, densely punctate, two pairs of eyes; lateral propodosoma with longitudinal to oblique granulated (dotted) striations, setae *sc*_1_ on prodorsal shield; opisthosomal striae transverse medially between setae *c*_1_-*f*_1_, except sub-medially changing to longitudinal to oblique between setae *f*_1_-*h*_1_, transverse posterior to *h*_1_, laterally with longitudinal to oblique irregular striations, all dorsal striae closely-spaced and lobed. Dorsal setae sub spautulate, serrtaed, short, not reaching to the setae next in line and not set on strong tubercles; Lengths of dorsal setae (without tubercles): *v*_2_ 27, *sc*_1_ 39, *sc*_2_ 31, *c*_1_ 32, *c*_2_ 37, *c*_3_ 23, *d*_1_ 22, *d*_2_ 35, *e*_1_ 21, *e*_2_ 26, *f*_1_ 16, *f*_2_ 22, *h*_1_ 34. Distances between dorsal setae *v*_2_-*h*_1_ 251, *v*_2_-*v*_2_ 41, *sc*_1_-*sc*_1_ 75, *c*_1_-*c*_1_ 67, *c*_1_-*c*_2_ 54, *d*_1_-*d*_1_ 32, *d*_1_-*d*_2_ 48, *e*_1_-*e*_1_ 36, *e*_1_-*e*_2_ 28, *f*_1_-*f*_1_ 17, *f*_2_-*f*_2_ 69, *f*_1_-*f*_2_ 28, *h*_1_-*h*_1_ 24.

**Figure 10.**
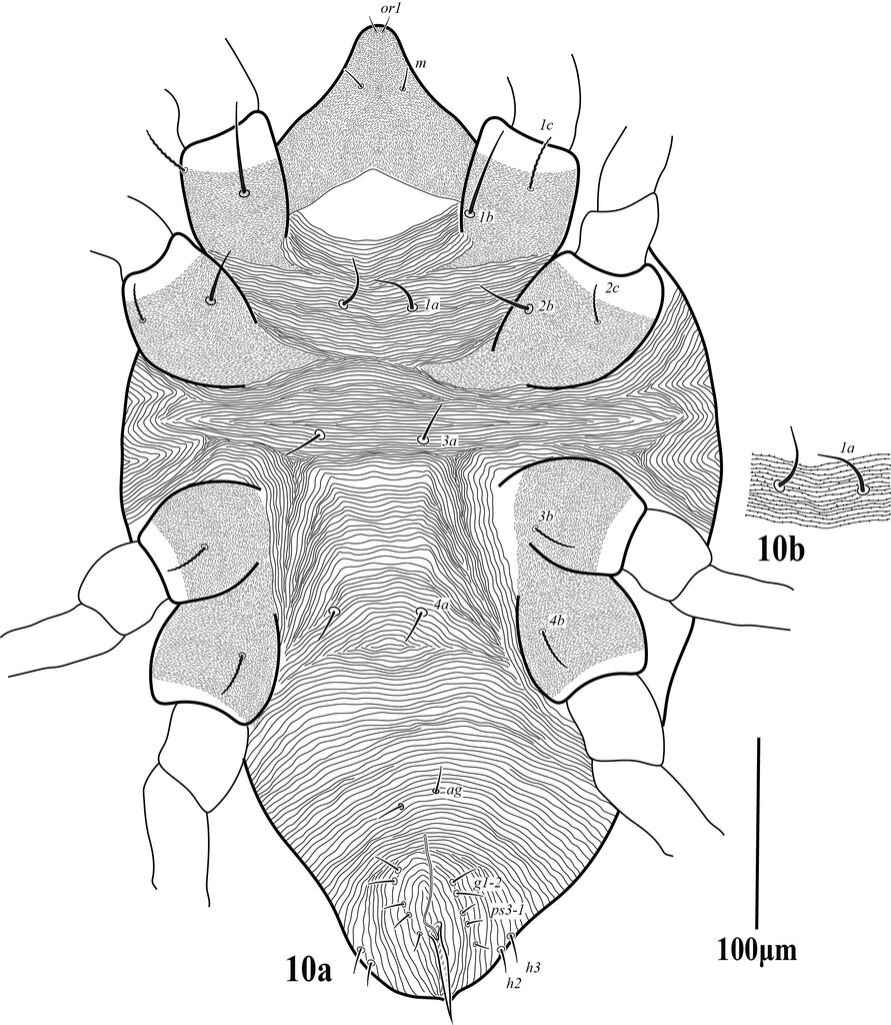
Petrobia (Tetranychina) cardi Chaudhri. Male, venter. Scale bar 100 μm.

**Venter** (Fig. 10). Integument between setae _1_a-_3_a with transverse lobed striae, between _3_*a* to _4_*a* with irregular oblique striae. Lengths of setae: _1_*a* 27, _1_*b* 24, _1_*c* 23, _2_*b* 23, _2_*c* 21, _3_*a* 21, _3_*b* 20, _4_*a* 18, _4_*b* 19, one pair of aggenital setae (*ag*) 11, two pairs of genital setae, _*g*_1 11, _*g*_2 12 and three pairs of anal setae (*ps*_1‒3_), *ps*_1_ = *ps*_2_ = *ps*_3_ = 9–10, *h*_2_ = *h*_3_ 11–12.

**Gnathosoma.** Ventral infracapitulum with one pair of adoral setae 8 and one pair of subcapitular setae *m* 16. Palp five-segmented (Fig. 12), palptarsus with one solenidion *ω* 7 (7‒8), three eupathia *ul''ζ* 8 (6‒8), *ul'ζ* 10 (9‒11), *sul* 9 (8‒10) and three setiform setae *a* 11 (9‒11), *b* 12 (12‒13), *c* 11 (10‒12); palp tibia with three setae *d* 19 (18‒22), *l*' 11 (11‒14), *l*'' 13 (12‒15) and one claw bifid distally, palp genu with one simple seta *l*'' 21 (19‒22) and palp femur with one serrated seta *d* 22 (20‒24) (similar to dorsal setae).

**Figure 11.**
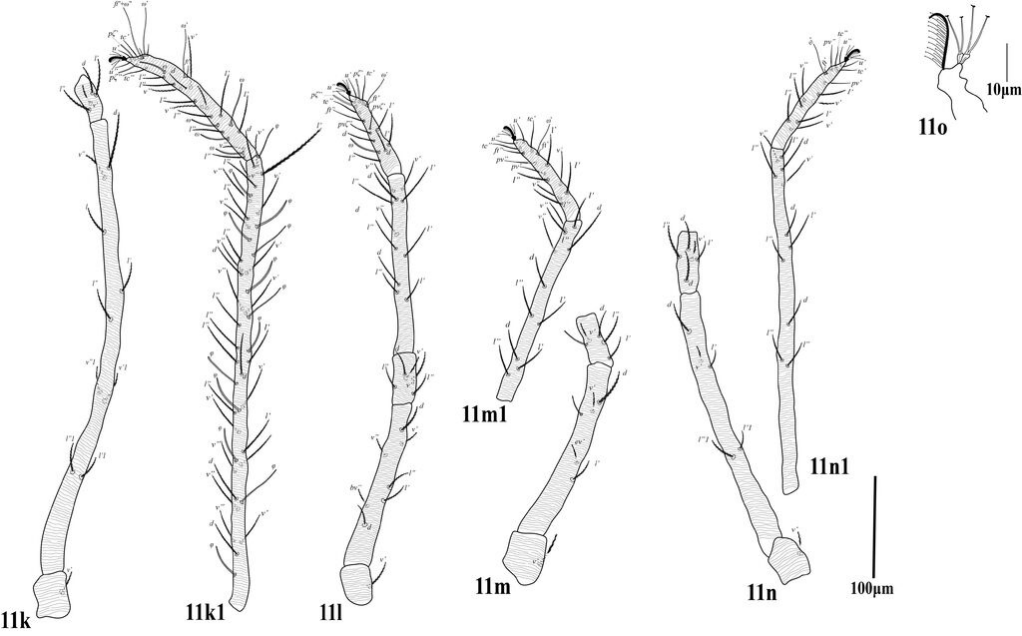
Petrobia (Tetranychina) cardi Chaudhri. Male. **k** Leg I; **11k** trochanter, femur; genu, **11k1** tibia, tarsus; **11l** Leg II; **m** Leg III **11m** trochanter, femur, genu; **11m1** tibia, tarsus; **n** Leg IV. **11n** trochanter, femur, genu. **11n1** tibia, tarsus. Scale bar 100 um. **11o** Empodium III. Scale bar 10 μm.

**Figure 12.**
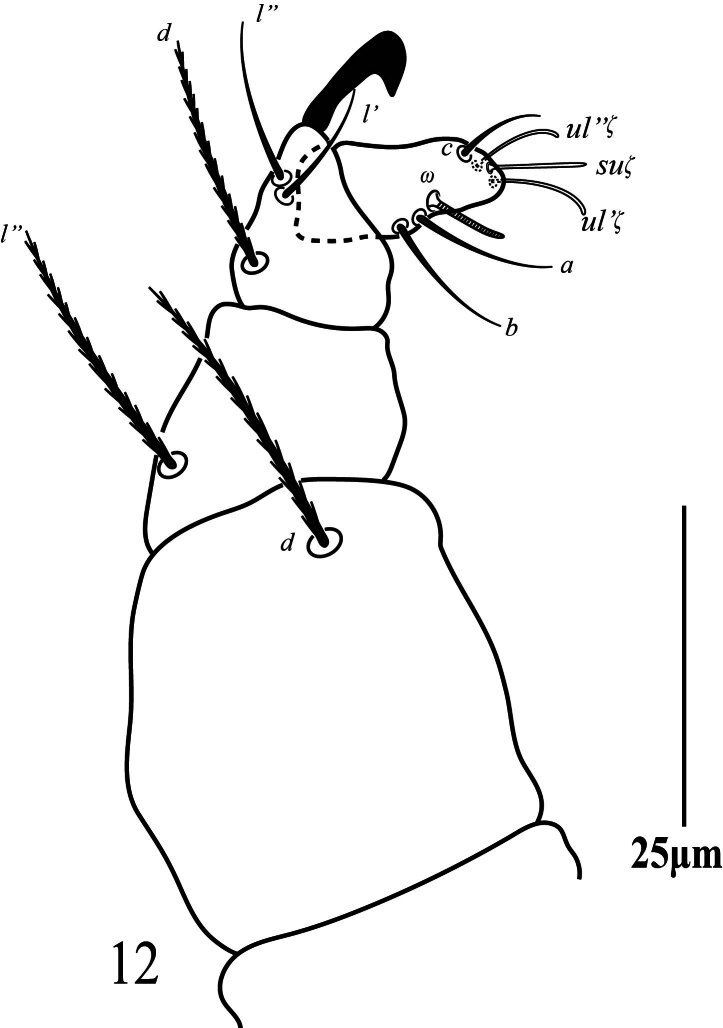
Petrobia (Tetranychina) cardi Chaudhri Male, Palp. Scale bar 25 μm.

**Legs** (Fig. 11). Length of leg I (trochanter to tip of tarsus) 1164, leg II 508; leg III 556; leg IV 820; length of leg segments, Leg I (trochanter 32, femur 461, genu 47, tibia 455, tarsus 169; leg IV trochanter 40, femur 270, genu 53, tibia 335, tarsus 122. Leg segment setal formula as follows: coxae 2-2-1-1; trochanters 1-1-1-1; femora 9-7-5-5; genua 4-5-5-5; tibiae 30+13φ+b (trichobothium-like seta) -11-11-11; tarsi 20 +5ω+3dup-14+1ω +1dup-13 +1dup-13 +1dup; solenidia on leg I Iω” 40, IIω” 41, IIω” 37, IIIω’ 15, IV ω’ 30, Empodia I–IV with true claws pad-like, empodial claws bearing two rows of ventrally directed hairs.

**Aedeagus**— Slightly directed ventrad, needle-like gradually tapering towards end.

#### Distribution

Abbottabad, Pakistan.

#### Remarks

The morphological characters of Petrobia (Tetranychina) cardi Chaudhri collected in the present study are almost similar to the original description (Chaudhri 1972) by leg chaetotaxy, shape of dorsal setae and peritremes, except some variations in the length of leg I 1220‒1293 vs. 1192 and leg IV 981‒1032 vs. 938 in the latter and length of dorsal setae.

## Supplementary Material

XML Treatment for Petrobia (Petrobia) pakistanensis

XML Treatment for Petrobia (Tetranychina) cardi
